# Neuroprognostication in unconscious patients after cardiac arrest: considering the time course of prognostic markers

**DOI:** 10.1016/j.aicoj.2026.100195

**Published:** 2026-10-29

**Authors:** S. Benghanem, E. Pruvost-Robieux, G. Hmeydia, H. Sellami, J. Mercier, M. Gavaret, T. Sharshar, A. Cariou, C. Calligaris, C. Legouy

**Affiliations:** a Medical ICU, Cochin Hospital, AP-HP, Paris, France; b University Paris Cité, Medical School, Paris, France; c After ROSC Network, Paris, France; d Institute of Psychiatry and Neuroscience of Paris (IPNP), INSERM U1266, Team Turc, Paris, France; e Neurophysiology and Epileptology Department, GHU Psychiatry & Neurosciences, Sainte Anne, Paris, France; f Department of Neuroradiology, G.H.U Paris Psychiatry & Neurosciences Paris, France; g Department of Neurocritical Care, G.H.U Paris Psychiatry & Neurosciences Paris, France; h Paris-Cardiovascular-Research-Center (Sudden-Death-Expertise-Center), INSERM U970, Paris, France

**Keywords:** Cardiac arrest, Coma, Disorder of consciousness, Electroencephalography, Imaging, Biomarkers, Evoked potentials, Neuroprognostication

## Abstract

Hypoxic–ischemic brain injury (HIBI) is a major determinant of outcome after cardiac arrest (CA) and accounts for approximately two-thirds of deaths in patients who remain comatose after return of spontaneous circulation (ROSC), frequently following withdrawal of life-sustaining therapy (WLST) based on an anticipated poor neurological prognosis. Accurate neuroprognostication is therefore crucial to identify patients with potential for neurological recovery while avoiding premature pessimistic predictions. Current guidelines recommend a multimodal approach integrating clinical examination, electrophysiology, biomarkers, and neuroimaging. However, neuroprognostication is inherently time-dependent: HIBI is a dynamic process, and the prognostic value of individual markers evolves substantially during the hours and days following CA. Some markers may provide relevant prognostic information early after ROSC, whereas others achieve optimal accuracy only after 24–72 h or later, as brain injury evolves and confounding factors progressively resolve. Thus, both the selection of prognostic markers and the timing of their assessment are critical to optimize prognostic accuracy. This review summarizes the temporal evolution of the prognostic performance of clinical examination, electroencephalography, somatosensory evoked potentials, biomarkers of brain injury, and neuroimaging after CA, and discusses how their integration according to optimal assessment windows may improve multimodal neuroprognostication and reduce prognostic uncertainty.

## Graphical abstract

**Figure:**
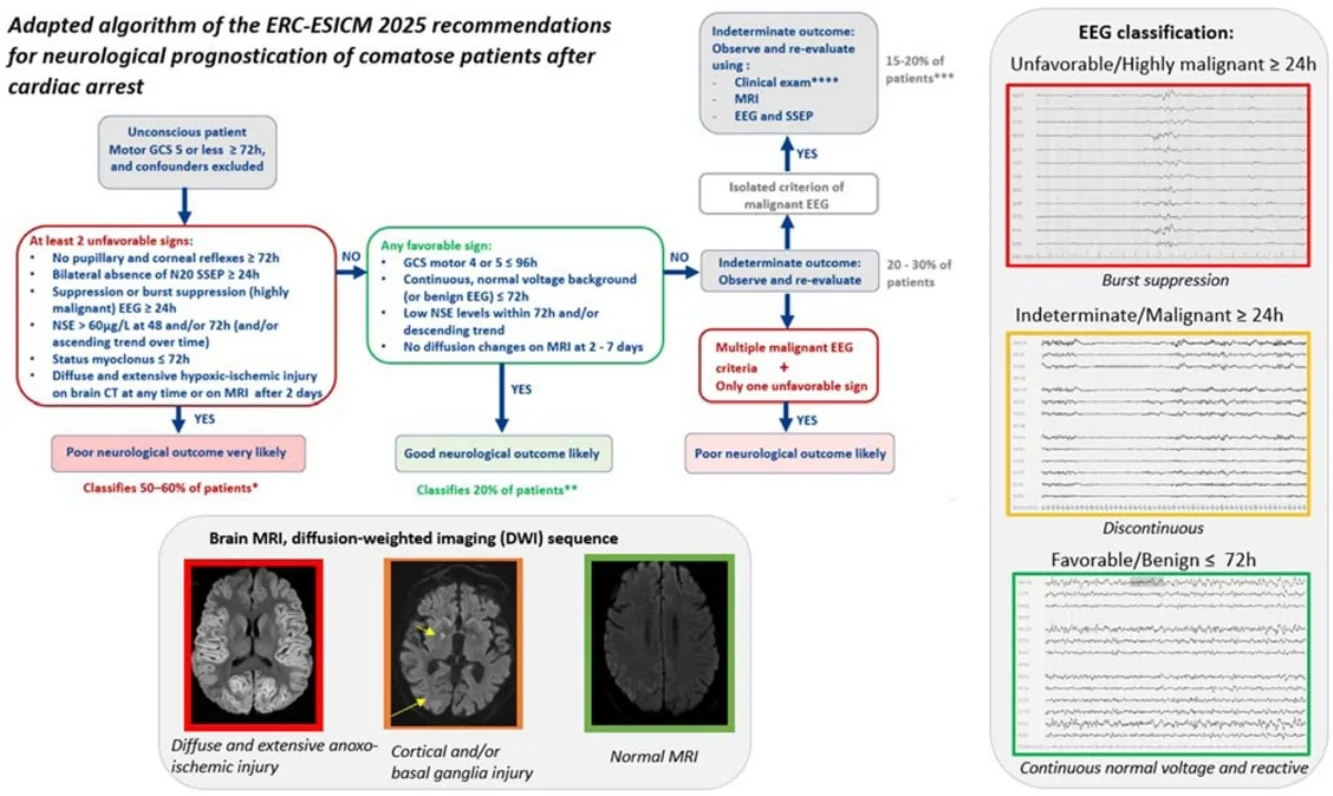

## Introduction

The neurological consequences of cardiac arrest (CA) are well established: the circulatory interruption can cause widespread and extensive brain damage, which can be exacerbated by ischemia-reperfusion mechanisms following the restoration of spontaneous circulation (ROSC). The first and most common consequence of this brain injury is loss of consciousness, explaining why most patients remain comatose and require intensive care. During the following hours and days, the subsequent course is variable, ranging from complete recovery within a few hours to a complete lack of neurological improvement indicating irreversible damage. Approximately two-thirds of deaths observed in unconscious patients are directly related to these hypoxic-ischemic brain injury (HIBI), most often due to withdrawal of life sustaining therapies (WLST) decisions made because of a neurological prognosis deemed too unfavourable [1,2]. For intensive care teams, the ability to reliably predict a favourable or unfavourable outcome is a major challenge, as it is both essential to pursue maximum therapeutic commitment when the hope of improvement persists, but also because reflection on the care plan is necessary when there is no longer any hope of improvement. The definition of a favourable outcome remains highly debatable, as the perception of an acceptable level of disability in daily life vary considerably according to individual values and beliefs [3]. Most recent studies define favourable neurological outcomes as a Cerebral Performance Category (CPC) score of 1–2 or a modified Rankin Scale (mRS) score of 0–3, corresponding to no or mild-to-moderate neurological disability. Conversely, a poor neurological outcome is generally defined by a CPC 3–5 or an mRS 4–6, encompassing severe neurological disability in conscious patients, persistent coma or vegetative state, or death [[4], [5], [6], [7]]. Importantly, neurological outcomes should ideally be assessed at least 3 months after CA, rather than upon ICU discharge, as some patients may continue to experience neurological recovery after ICU discharge [7,8].

Considerable efforts have been made in recent years to clarify the respective performance of the markers that can be used in this situation, leading to the recommendation of a multimodal strategy combining different clinical and paraclinical indicators [7]. The prognostic value of this strategy depends not only on the proper mastery of the indicators used but also on adherence to the timeframes for their collection, as their prognostic value varies according to the time elapsed since CA and the onset of acute brain injury. The present review aims to provide a comprehensive overview of the temporal evolution of neuroprognostication markers after CA—including clinical examination, biomarkers, electroencephalography, somatosensory evoked potentials, and neuroimaging—and to highlight the importance of adhering to recommended assessment timelines to ensure optimal prognostic accuracy.

## Time dependence of hypoxic ischemic brain injury

HIBI develops through a dynamic process that unfolds over time following CA. The initial phase begins with the abrupt cessation of cerebral blood flow, resulting in global cerebral ischemia and oxygen deprivation. This primary insult rapidly leads to energy failure, loss of ionic homeostasis, cellular membrane dysfunction, and neuronal injury. Following ROSC, a secondary phase of ischemia–reperfusion injury occurs during the first hours after resuscitation [2,6]. This stage is characterized by excitotoxicity, oxidative stress, neuroinflammation, endothelial dysfunction, microvascular abnormalities, blood–brain barrier disruption, and cerebral oedema, all of which contribute to the progression of brain injury. In the subsequent hours and days, secondary brain injury may be further aggravated by systemic insults such as arterial hypotension, hypoxemia, dysregulated carbon dioxide levels, fever, electrolyte disturbances, or dysglycemia [2,6]. Over the following weeks and months, the injured brain undergoes a phase of remodelling during which competing restorative and degenerative mechanisms coexist. On the one hand, neuroplasticity, synaptogenesis, and, to a lesser extent, neurogenesis may promote functional recovery and neurological improvement. On the other hand, persistent neuroinflammation and delayed neuronal and glial apoptosis may contribute to progressive tissue loss and cerebral atrophy, which can sometimes be detected on follow-up neuroimaging. Because different brain regions exhibit varying susceptibility to ischemic and reperfusion injury, the extent and distribution of damage are heterogeneous, resulting in a broad spectrum of neurological outcomes ranging from complete recovery to severe disability, prolonged disorders of consciousness, or brain death [2,6].

## Defining prognostic accuracy

When assessing the prognostic value of a marker, particular attention is paid to its specificity, sensitivity, and false-positive rates (FPR). When predicting poor outcomes, specificity is prioritized to minimize the risk of false-positive predictions. A marker is considered highly specific for poor neurological outcome when its specificity exceeds 95%, corresponding to a false-positive rate <5% [7]. However, such high specificity inherently comes at the expense of sensitivity, which is often low to moderate [5]. This explains why some patients may experience poor neurological outcomes despite the absence of poor-outcome predictors according to the 2025 ERC–ESICM guidelines [6,9,10]. Conversely, when predicting favorable outcomes, both sensitivity and specificity should be considered and an optimal balance between the two should ideally be sought for quantitative markers, such as NSE cut-off values [4]. Importantly, the interpretation of prognostic accuracy must also account for potential methodological biases. Most deaths following CA occur after WLST for neurological reasons, raising concerns about self-fulfilling prophecy bias: indeed, prognostic test results may directly influence WLST decisions and patient outcomes. Nevertheless, studies conducted in countries where WLST is not routinely practiced for cultural or religious reasons have reported comparable prognostic performance to that observed in countries where WLST is commonly considered, suggesting that this bias may not fully explain the predictive accuracy of these markers [11].

## Neurological examination

About 80% of patients with ROSC following CA are comatose at ICU admission and nearly 50% of survivors are still unconscious at 72 h [6]. Most patients with a favourable outcome begin to recover consciousness a few hours after cessation of sedation [12]. According to the ERC–ESICM guidelines, awakening is defined as the ability to reproducibly follow verbal commands, corresponding to a motor score of 6 on the Glasgow Coma Scale (GCS) [7]. Nevertheless, delayed awakening is not uncommon after sedation weaning. In some cases, awakening may be observed up to 12–25 days after ROSC [13]. Recognized risk factors for delayed awakening include post-resuscitation shock, acute renal insufficiency, older age, and use of long-acting (e.g., midazolam) vs. short-acting (e.g., propofol) sedative agents [12,14,15].

The neurological state of consciousness after CA is highly heterogeneous and subject to time-related variations. A complete physical examination using adapted scales and rigorous definitions is recommended to accurately assess the neurological state and predict recovery of consciousness [16,17]. Thus, clinical examination remains a cornerstone of neuroprognostication, although it is subject to important limitations. To avoid underestimating the level of consciousness, assessments should only be performed after adequate clearance of sedative agents. Any potential confounders that could lead to superimposed encephalopathy must be systematically identified and corrected. These include fever, renal or hepatic dysfunction, electrolyte imbalances, antiepileptic drug toxicity, and exposure to neurotoxic agents such as neuroleptics or antibiotics [7]. Additionally, fluctuations in the neurological status of brain-injured patients, as well as inter-observatory variability, should be carefully considered. Several clinical parameters should be systematically collected, including: 1/ brainstem reflexes (particularly the pupillary light reflexes - PLR and corneal reflexes), 2/ early myoclonus or status myoclonus occurring in the first days after CA, 3/ motor response to stimuli, as assessed by the Glasgow Coma Scale (mGCS), and 4/ patient’s level of consciousness using appropriate validated scales, such as Coma Recovery Scale Revised (CRS-r) [18]. Because these different clinical data can fluctuate over time, repeated assessments are required, ideally multiple times per day.

During the early phase (i.e., usually within the first 7 days), the literature provides relatively abundant data on clinical prognostic markers. During this period, brainstem reflex monitoring is essential. While the bilateral absence of PLR or corneal reflexes demonstrates low specificity and high false-positive rate (FPR) for prediction of poor outcome within the first 24 h post-CA, the FPR decline after 24−72 h. A combined bilateral absence of both the PLR and corneal reflexes after 72 h has 100% specificity in predicting an unfavourable outcome, despite having a sensitivity of only 20% [5,7]. Using pupillometry to quantitatively assess the PLR may improve accuracy, particularly when pupils are pinpoint. Automated pupillometry enables an objective assessment of the PLR by providing a Neurological Pupil index (NPi) ranging from 0 (non-reactive) to 5 (normal). Although the optimal NPI threshold is still being debated, an NPI of less than 2/5 after 72 h seems to indicate a poor outcome with high specificity, despite limited sensitivity of around 20−35% [19,20]. Regarding abnormal movements, early isolated myoclonus (i.e. brief, sudden muscular contractions affecting muscle segments, individual muscles, or muscle groups) observed in the first 72 h is associated with unfavourable outcome in most cases. Nevertheless, according to studies, the FPR ranges from 0 to 22%, suggesting that this criterion is not a robust marker for predicting a poor outcome, due to the risk of making pessimistic predictions that are not accurate [5]. Conversely, early-onset *status myoclonus* (defined as generalized, continuous myoclonic movements persisting beyond 30 min) demonstrates nearly 100% specificity when observed within the first 72 h, although it may be masked by sedation [5]. The prognostic value of the motor component of the GCS score for predicting poor outcomes is limited. Thus, the 2025 ERC-ESICM guidelines recommend combining it with at least two other markers of poor outcome [7]. Nevertheless, a motor score of less than 4 (i.e., absent motor response, decerebration, or decortication) correlates with an unfavourable outcome. Conversely, scores of 4 (i.e., flexion withdrawal) or 5 (i.e., localization to pain) when observed within the first 96 h after CA may indicate a favourable outcome [4,7,9].

Beyond the early phase (i.e., usually after day 7), there is still limited prognostic data for CA patients. However, the bilateral absence of both PLR and corneal reflexes at this time appears to be highly specific for prediction of unfavourable outcomes [5]. Late-onset myoclonus, which appears after a symptom-free interval of more than 72 h after CA and is typically triggered by spontaneous movements (*action myoclonus*) in patients recovering consciousness, is consistent with Lance-Adams syndrome [21,22]. Lance–Adams syndrome is usually treated through gradual escalation of anti-myoclonic therapy (e.g., levetiracetam, perampanel, and/or sodium valproate), and requires an evaluation by a specialized expert team. Although Lance–Adams syndrome is not generally considered a poor outcome, its most severe forms, characterized by persistent involuntary movements despite appropriate treatment, may substantially impair functional independence [21]. Regarding the level of awareness, patients may recover arousal (i.e., eyes opening) after the initial period of coma, without showing evidence of consciousness. These entities are usually referred to as disorders of consciousness (DoC). They include the unresponsive wakefulness syndrome (UWS) and the minimally conscious state (MCS). Both conditions are characterized by preserved brainstem-mediated arousal and persistent dysfunction of the supratentorial networks that support consciousness. It is critical to distinguish between these DoC subtypes, as patients in MCS are significantly more likely to regain consciousness and have a better functional outcome after 1 year than those in UWS [23]. For these reasons, daily clinical assessments for emerging signs of consciousness are recommended [16,17]. The CRS-r is the most widely used and sensitive tool for classifying patients as UWS (i.e., eye opening without environmental interaction), MCS (i.e., minimal interaction without functional communication), or conscious (i.e., reliable communication code established) (ESM - Table S1) [18]. To maximize the detection of consciousness signs, the clinical examination should be conducted in a distraction-free environment. Prior stimulation of the patient should be used to achieve optimal arousal and attention. The observations of caregivers and family members should also be considered [24]. Indeed, stimulus familiarity may be particularly relevant in patients with DoC [25]. Likewise, assessing command-following using instructions delivered in the patient’s native language is strongly recommended in order to minimize the risk of misclassification of the patient’s level of consciousness. Late awakening (i.e., after 14 days) with favourable outcome is rare but documented [13,[26], [27], [28]]. Nevertheless, a prolonged time to recovery of consciousness seems to be correlated with an increased risk of neurological cognitive sequelae [13,15,26,28]. Finally, a persistent coma or UWS without confounding factors at 25 days or more than one month after CA substantially diminishes the possibility of neurological recovery [27,29].

## Electroencephalogram (EEG)

### Prognostic value of EEG patterns

It is recommended to wait at least 72 h before beginning to analyse the indicators used to assess the prognosis of a comatose patient. However, it is crucial to include prognostic markers obtained during the first few hours following CA—such as electroencephalograms (EEGs)—in this analysis. EEG findings are typically categorized according to background, superimposed abnormalities (epileptiform discharges also called rhythmic or periodic patterns, or RPPs), and reactivity (ESM - Fig. S1). Regarding background activity, the severity of HIBI is correlated with the predominant frequency, amplitude and continuity. It ranges from slow background activity (theta or delta frequency), to low voltage (10μV < amplitude<20μV the majority of time), sub-continuous (amplitude <10μV, during 0–10% of the recording), discontinuous (amplitude <10μV, during 10–49%), burst suppression (amplitude <10μV, during 50–99 %) or suppression background (amplitude <10μV all the time). RPPs, electroencephalographic seizures or unreactive EEG could also reflect HIBI, with varying degrees of severity. Regarding classification, the American Clinical Neurophysiology Society (ACNS) critical care EEG terminology [30] was adapted by Westhall et al. [31], resulting in three main categories: highly malignant, malignant and benign EEG. This classification is now the most commonly used for prognostication after CA [4,[32], [33], [34], [35], [36], [37], [38], [39], [40]]. This enables standardized interpretation by classifying each tracing into one of the following three EEG categories (Table 1):

• **“Unfavorable” EEG patterns (historically called “Highly malignant patterns”),** including 3 main patterns: *suppression, suppression with superimposed periodic discharges,* and *burst suppression*. These patterns are recognized as robust markers of poor outcome (i.e., specificity 95–100 %, FPR 0−5%, sensitivity highly heterogeneous according to timing of EEG recording) when observed at least 24 h after CA [5,7,38]. Therefore, these profiles are considered one of the six prognostic markers of poor prognosis in the 2025 ERC-ESICM guidelines [7]. The morphology of bursts in burst-suppression patterns can also be very informative. Burst-suppression with identical bursts (BSIB) is characterized by highly stereotyped bursts with nearly identical morphology, whereas burst-suppression without identical bursts exhibits greater variability in burst morphology. BSIB appears to be more specifically associated with severe HIBI, while non-identical burst-suppression may also result from high doses of sedative agents. After CA, BSIB is strongly associated with poor outcomes, and its prognostic accuracy appears less dependent on EEG timing than that of non-identical burst-suppression [41].
• **“Indeterminate” EEG patterns (historically called “malignant patterns”)** include *abundant and generalized rhythmic or periodic patterns (RPPs), electroencephalographic seizures, discontinuous background, low voltage, unreactive EEG* and *antero-posterior gradient reversion*. Most studies assessed the prognostic value of these patterns as a whole, rather than evaluating their individual performance [5,37]. The prognostic value of “indeterminate” EEG remains very heterogeneous among the different EEG patterns that can be observed. According to studies, the FPR for predicting a poor outcome ranges from 0 to 33%. Thus, these EEG are not recognized as robust markers of poor outcome, due to the risk of false pessimist prediction.
• **“Favourable” EEG patterns (historically called “benign patterns”)** are defined as *absence of malignant or highly malignant features*, corresponding to a *continuous or nearly continuous background, normal voltage, no superimposed abnormalities* and *preserved reactivity to stimuli*. A “favourable/benign” EEG is highly predictive of good outcome when recorded during the first 12, 24–72 h after CA (specificity 56.5–100 %, predictive positive value - PPV 70–94 %, sensitivity 30–80 %) [31,36,40,[42], [43], [44], [45], [46]]. A major point is that, in neuroprognostication after CA, the background is the most important component of the EEG. Thus, early continuous or nearly continuous, with normal voltage and no superimposed abnormalities (especially within 12−24 h or even in the first 72 h) is now one of the four prognostic markers used to predict a good outcome in the 2025 ERC-ESICM recommendations [7].

**Table 1:** Definitions and prognostic value of each EEG patterns (adapted from ACNS terminology and Westhall et al classification).

EEG	Patterns	Definition	False positive rate (%) CI95 (%) for poor outcome prediction
**“Unfavorable/highly malignant” EEG ≥24 h after CA –*****poor outcome likely if used in a multimodal approach***			
Including	Suppression	Suppressed background (<10μV) during all the recording, no discharge	0% CI95(0−11.7)
	Suppressed background + periodic discharges	Suppressed background (<10μV) during all the recording, with periodic discharges	0% CI95(0–23.1)
	Burst suppression	Alternance of: - suppressed background (suppression period >50% and <99%of the recording) - identical bursts Or alternance of: - suppressed background (suppression period >50% and <99%of the recording) - non-identical bursts	Identical burst: 0% CI95(0–20.6); Non identical burst: 0−1.4% CI95(0−3.1)
A. “Unfavorable” EEG ≤ 24 h after CA (regardless of the pattern); B. “Unfavorable” EEG ≥24 h after CA (regardless of the pattern); C. “Unfavorable” EEG ≥24 h and hypothermia*; D. “Unfavorable” EEG ≥24 h and normothermia*; E. “Unfavorable” EEG ≥24 h with ongoing sedation*; F. “Unfavorable” EEG ≥24 h with sedation stopped*; G. "Unfavorable” EEG ≥24 h and unreactive background*			A. 0−14.3% CI95(0–58); B. 0−6.8% CI95 (0–10.3); C. 0−8.8% CI95 (0–14.2); D. 0−4.2% CI95 (0–9.0); E. 0−7.4% CI95 (0–12.7); F. 0−7.4% CI95 (0–16.7); G. 0−3.1% CI95 (0–5.8)
**“Intermediate/Malignant” EEG ≥ 12 h after CA –*****indeterminate outcome***			**False positive rate (%) CI95 (%) for poor outcome prediction**
Malignant periodic or rhythmic patterns	Periodic discharges Also called epileptiform features	Abundant non-evolving* and low frequency (0.5−2.5 Hz) periodic discharges (spike, sharp or wave) during >50% of the recording No suppressed background	Early (< 24 h after CA): 0−3% CI95(0−34.8); Late (≥ 24 h after CA): 0–33.3 % CI95(0−70.1)
	Rhythmic discharges Also called epileptiform features	Abundant non-evolving* rhythmic discharges during >50% of the recording No suppressed background	Early (< 24 h after CA): 0−3% CI95(0−34.8); Late (≥ 24 h after CA): 0–33.3 % CI95(0−70.1)
	Unequivocal electrographic seizure or electrographic status epilepticus	- Epileptiform discharges >2.5 Hz for ≥10 seconds or - any pattern with definite temporo-spatial evolution and lasting ≥10 seconds Electrographic seizures for ≥10 continuous minutes or for a total duration of ≥20% of any 60-minute period of recording	0−17.4% CI95(0−26.7)
Malignant background	Discontinuous background	Suppression periods (<10μV) or low voltage (10μV < ampl <20μV) constituting >10% but <49% of the recording	0−13.8% CI95(0.1-48.2)
	Low voltage	10μV < Amplitude < 20μV	0−12.1% CI95(0−29.2)
Unreactive background	Unreactive EEG	Absence of reproductible modification for both sound, visual and sensory stimuli	0−50% CI95(0−70.9)
Isolated vs multiple “intermediate/malignant” criteria ≥ 24 h after CA (among unreactive, low voltage, discontinuous or rhythmic-periodic discharges)**			**False positive rate (%) CI95 (%) for poor outcome prediction**
One criterion of “intermediate malignant” EEG**			18.61% CI95 (14.33–22.90) or 52% CI95 (34–69)
Two criteria of “intermediate/malignant” EEG**			1.89% CI95 (0.39–3.39) or 4% CI95 (1–18)
Three criteria of “intermediate /malignant” EEG**			0% CI95 (0−0)
Four criteria of “intermediate /malignant” EEG**			0% CI95 (0−0)
**“Favourable/Benign” ≤ 72 h after CA –*****good outcome likely***			**Specificity (%) CI95 (%) for good outcome prediction**
Including	Continuous normal-voltage background without discharges Or Nearly continuous normal-voltage background without discharges and reactive EEG	Continuous: Continuous background during all the recording Nearly continuous: Suppression periods (<10μV) constituting <10% of the recording Reactive EEG: Change in cerebral EEG activity to stimulation, include change in voltage or frequency (including attenuation of activity)	Continuous/nearly continuous: 56.5 to 100% CI95(45.3−100); Reactive EEG: 57.1 to 85% CI95(37.2−93)

*Non-evolving: no temporal or spatial evolution. CI: confidence interval; EEG: electroencephalogram. Data presented in this table are mainly derived from two systematic reviews by Sandroni et al. on predictors of poor neurological outcome (Intensive Care Medicine, 2020) and favorable neurological outcome (Intensive Care Medicine, 2022), as well as the 2025 ERC–ESICM guidelines on post-resuscitation care (Nolan et al., Intensive Care Medicine/Resuscitation, 2025). *According to Turella et al (Intensive Care Medicine, 2024). **According to Benghanem et al (Neurology 2025) and Westhall et al (Neurology 2016).

Thus, many patients with an “**Indeterminate/malignant” EEG patterns** remain in prognostic uncertainty. Nevertheless, the prognostic implications of a single malignant EEG feature are likely to differ from those associated with multiple concomitant malignant EEG features in terms of the severity of HIBI. A bicentric study including 1076 patients assessed the prognostic value of combined malignant EEG criteria observed on a single early EEG towards poor outcome and evaluated the effect on prognostic uncertainty. A single malignant EEG feature had limited specificity (81.4%) for predicting poor outcome. Conversely, the presence of 2, 3, or 4 malignant EEG criteria was highly indicative of poor outcome (specificity of 98.1%–100%). Finally, multiple malignant EEG features reclassified approximately 31% of patients from the “*indeterminate*” category to the "*likely poor outcome*" category, substantially reducing prognostic uncertainty [37]. The same results were found in a previous study [31]. Nevertheless, these criteria should always be considered as a part of a multimodal approach, to minimize the risk of falsely pessimistic predictions. Finally, the presence of an isolated malignant criterion should prompt clinicians to observe and re-evaluate the patient, as neurological recovery remains possible, according to guidelines [7].

This new EEG-based approach may significantly reduce prognostic uncertainty in this population. Indeed, the current algorithm for predicting poor outcome - which includes “unfavorable/highly malignant” EEG patterns > 24 h - appears to be very effective, classifying 50–60% of patients as *“poor outcome very likely”* [9,10]. Conversely, criteria predictive of a favourable outcome - including “favourable/benign” EEG patterns < 72 h - allow for the reclassification of approximately 20% of patients who were initially considered with *“indeterminate prognosis”* [36]. These favourable prognostic criteria have been extensively validated [4,9,34,36,40,47] and have now been incorporated into the 2025 ERC–ESICM guidelines [7]. Consequently, the proportion of patients with prognostic uncertainty could be reduced to only 20–30%. Within this subgroup, using multiple “indeterminate/malignant” EEG criteria could reclassify about a third of patients from *“indeterminate prognosis”* to *“poor outcome likely”* [37] (Fig. 1). However, these “multiple indeterminate/malignant” EEG criteria are not currently included in ERC-ESICM recommendations [7], as their prognostic performance requires further external validation before they can be recommended for routine clinical practice. Finally, future research aimed at identifying and validating new prognostic markers will likely focus primarily on this subgroup of patients with an indeterminate outcome.

**Fig. 1:**
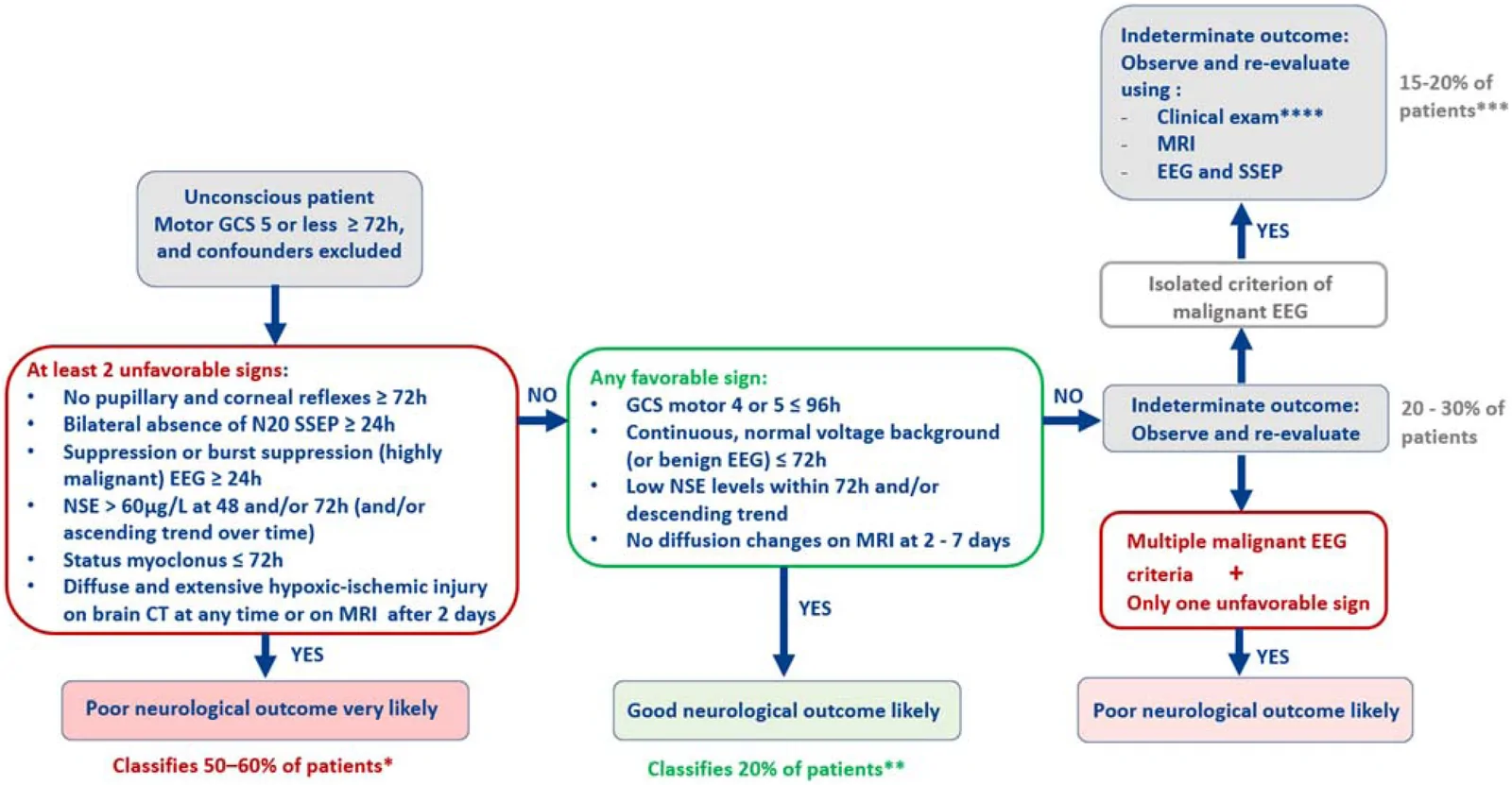
**Modification of the 2025 ERC-ESICM algorithm to include tentative prognostic strategies in patients with indeterminate outcome (Nolan et al, ICM 2025)**. Status myoclonus ≤72 h: Generalized, continuous myoclonic movements persisting for more than 30 min. For pupillary assessment: Automated pupillometry should be considered when available, a Neurological Pupil Index (NPi) <2/5 being highly specific for poor neurological outcome. Low NSE level within 72 h: NSE <17 μg/L (according to Sandroni et al ICM 2022, Lagebrant et al Resuscitation 2025, and Besnard et al Critical Care 2025) or NSE < 30 or 40−45 μg/L (according to Vanat et al Critical Care Medicine 2023, Besnard et al Critical Care 2025). Regarding NSE trends, increasing NSE levels between 24, 48 and 72 h after CA are strongly associated with poor outcomes, whereas decreasing levels are associated with favorable outcomes. The absolute NSE level at 24 h does not provide robust prognostic information. *Performance of the algorithm for poor outcome prediction according to Bougouin et al Resuscitation 2024 & Lagebrandt et al Resuscitation 2025; **Performance of the algorithm for good outcome according to Besnard et al Critical Care 2025. ***The use of multiple vs isolated malignant EEG criteria may reduce the prognostic uncertainty by 30% according to Benghanem et al Neurology 2025. ****The persistence of coma or unresponsive wakefulness syndrome (UWS) beyond 25 days (according to Hayamizu et al Resuscitation 2023) or 1 month (according to Petzinka et al Resuscitation 2018) after CA, in the absence of confounding factors, markedly reduces the probability of a favorable long-term outcome; conversely, a MCS or exit-MCS should prompt clinicians to wait and reassess the patient (according to Rohaut et al, Nature Medicine 2024).

### Time effect on the prognostic value of EEG patterns

The timing of the EEG recording varies considerably between studies. It ranges from 6−12 h (under sedation) to 7 days or more [4,5] (Table 1). As EEG reflects “real time” cortical activity, patterns of evolution over time could provide interesting prognostic information(Fig. 2). However, both the timing of EEG recording and the effects of sedation, commonly administered during the first 24 h after CA, must be carefully considered. When the EEG is performed immediately after ROSC, the EEG background is sometimes suppressed, even in some patients who will subsequently experience early awakening after sedation is stopped. For this reason, the EEG pattern should not be used for predicting poor outcome within the first 12 or even 24 h after CA, due to the risk falsely pessimistic prediction. After 12−24 h, it typically evolves rapidly towards different “unfavourable”, “indeterminate” or “favourable” patterns, highly correlated with the severity of the HIBI and the neurological outcome.

**Fig. 2:**
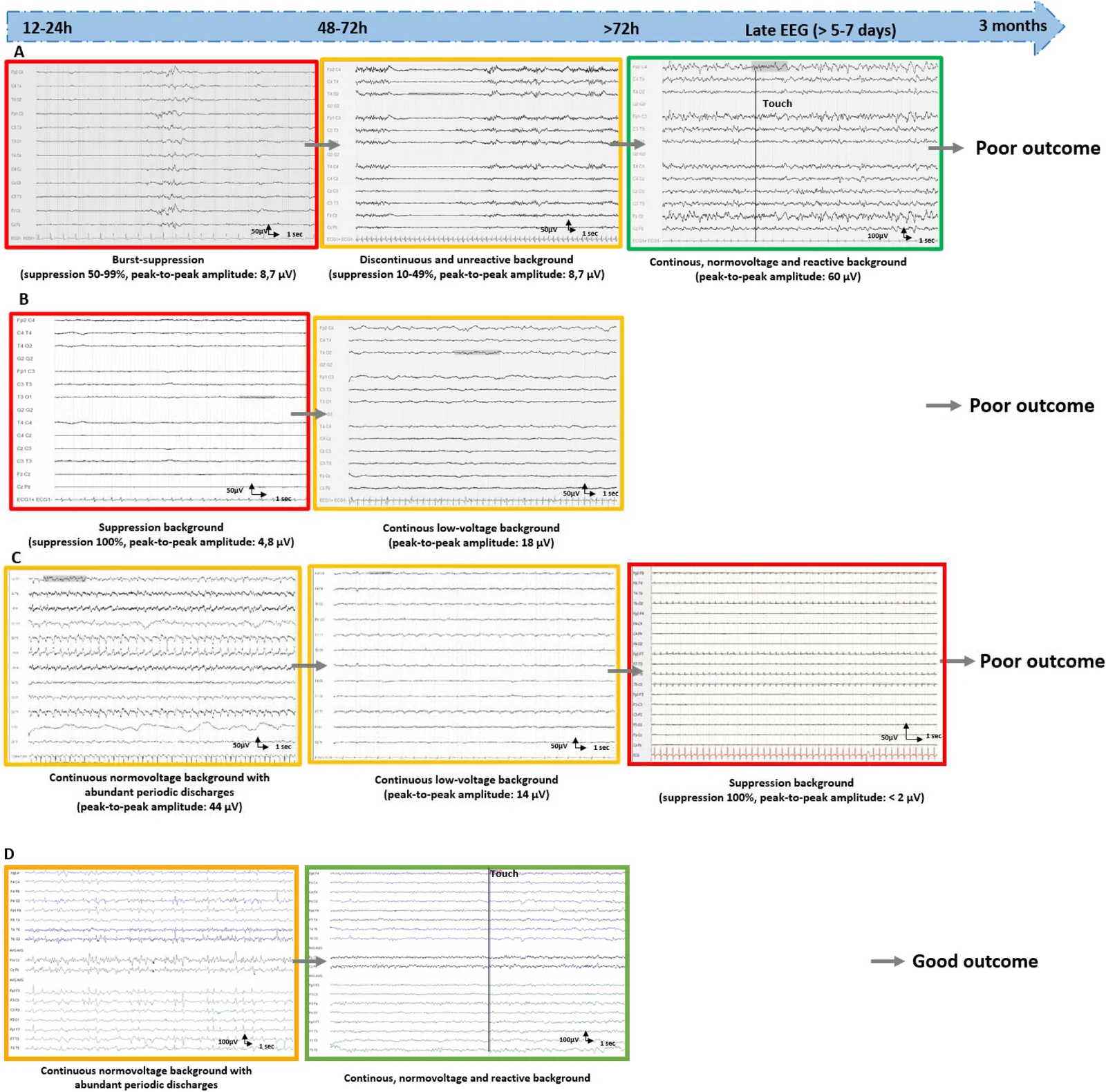
**Examples of the evolution of EEG patterns over time in comatose patients after cardiac arrest. EEG categories are color-coded as follows: red indicates highly malignant patterns, orange malignant patterns, and green benign patterns. In these four patients, EEG was performed at different time point**: **(A) Patient 1**: EEG performed in the first days following CA revealed a « highly malignant » pattern with burst-suppression. A second EEG performed a few days later showed a “malignant” pattern (discontinuous and unreactive background) and a last EEG, performed more than 72 h after CA, revealed a “benign” pattern according to Westhall et al (Neurology 2016) and ACNS terminology (J Clin neurophysiol 2021), with a continuous, normal voltage and reactive background. The patient had a poor neurological outcome at 3 months. **(B) Patient 2**: early EEG revealing a “highly malignant” pattern with a suppression background; the second EEG became “malignant” (continuous, low-voltage and unreactive background). The patient had a poor neurological outcome at 3 months. **(C) Patient 3**: early EEG at 24 h post-CA revealed a “malignant” pattern (continuous and normal voltage background with abundant periodic discharges, also called rhythmic periodic patterns - RPPs); the continuous EEG monitoring showed a gradual disappearance of periodic activities between 24−48 h post-CA but the persistence of a low-voltage background (“malignant pattern”) and then a suppressed background (“highly malignant pattern”); the patient ultimately died (brain death). **(D) Patient 4**: early EEG at 24 h CA revealed a “malignant” pattern (continuous and normal voltage background with abundant periodic discharges - RPPs); the second EEG was “benign (continuous, normal voltage and reactive background) in the first 72 h after CA; the patient had a good outcome at 3 months (For interpretation of the references to colour in this figure legend, the reader is referred to the web version of this article).

Regarding poor outcome prediction, the timing of EEG recording is a major issue. Indeed, “unfavorable” EEG patterns tend to disappear in the first days after CA, which reduces their sensitivity for predicting a poor outcome [5,33,43,48,49]. Thus, “unfavorable” EEGs, recorded as early as 6−12 h after CA, have the greatest sensitivity (71–91% according to studies), despite moderate specificity. This sensitivity becomes more variable (10–84%) between 12 and 24 h, then decreases sharply between 48 and 72 h and beyond (6–48%). Moreover, in a large prospective cohort study, the sensitivity of a “unfavorable” EEG was 47%, 30% and 22% at 12, 24 and 36 h after CA respectively, with a very high specificity in all cases [50]. Thus, some patients with an unfavourable outcome may initially present an “unfavorable” EEG at 12−24 h (suggesting a “*poor outcome likely*”) which then evolves toward an “indeterminate” EEG (in favour of an “*undetermined outcome*”) (Fig. 2). These findings should encourage intensivists to perform EEG recording as early as 24 h after CA - ideally in the absence of sedation or with the minimal effective dose - in order to assess long term neurological prognosis, and to repeat the EEG at 48−72 h in the absence of favourable clinical evolution [7]. Finally, data regarding “unfavorable” EEG performed beyond 72 h are more limited and less robust. Nevertheless, it may be highly specific of poor outcome, despite a very low sensitivity [51].

Conversely, in the vast majority of patients with an early awakening and a good outcome at 3 months, EEG background activity becomes continuous or sub-continuous, of normo-voltage and reactive within the first 12, 24 or 72 h after ROSC, even with low to moderate dose of sedation. Thus, the early recording of this pattern is considered as highly specific of a good outcome. Interestingly, the prognostic value of a “favourable” EEG recorded at 12−24 h after CA was higher (specificity 86–95 %) compared to a “favourable” EEG observed at 72 h (specificity 65–78 %) [4,44,52]. Data regarding “favourable” EEG observed beyond 72 h are more limited [4]. At this stage, EEG background activity tends to be more continuous in all patients (including those with poor outcome at 3 months), which probably reduces the specificity of this EEG criterion for predicting a good outcome. Thus, the prognostic value of this “late favourable” EEG appears to be limited and, in all case, inferior to that of “early favourable EEG” (i.e., first 24−72 h after CA).

### Sedation effect on EEG

Sedation is often used in the first 24 h after CA, to facilitate post-resuscitation care (mechanical ventilation, management of post-resuscitation shock, temperature management) but it can alter the prognostic assessment in different ways. Sedation may delay awakening [6,14], confound pupillary and corneal reflexes and alter EEG patterns [53,54]. These different points encourage the use of light to moderate dose of sedation with short-acting drugs, such as propofol [7,12]. The question of whether EEG remains a reliable prognostic tool in sedated patients has been debated for a long time. Most sedative drugs have similar effects on EEG spectrum, namely decreasing frequency, continuity and amplitude, in a dose dependent manner. More specifically, high doses of midazolam and propofol could generate burst suppression patterns, which is an important predictor of unfavourable outcome [7]. Nevertheless, the induction of a burst suppression pattern is usually observed with higher doses than those used for the management of post-CA patients [48,55,56]. Thus, an increasing amount of data suggests that light to moderate sedation (i.e., median doses < 3.0–3.5 mg/kg/h of propofol or midazolam < 63–68 μg/kg/h) does not affect the prognostic value of EEG. Moreover, patients who displayed a continuous background seems to be sedated with higher doses (2.67 mg/kg/h of propofol) compared to patients with burst suppression or suppression EEG patterns (2.07 mg/kg/h and 1.94 mg/kg/h respectively) [48]. These results, with some others [38,44,49,55,[57], [58], [59], [60], [61], [62], [63]], suggest that light to moderate sedation used of the post-CA management does not significantly impair the prognostic accuracy of the early EEG (i.e., at 12−24 h after CA) compared to recordings carried out after 48−72 h. Thus, EEG should ideally be performed in the absence of sedation or under the lowest effective sedative dose, as early as 24 h after CA to avoid missing the early time window during which EEG provides the highest prognostic accuracy. Caution is needed when patients required deep sedation with two different drugs, as the association of midazolam and propofol decreased the probability to detect “favourable” EEG patterns [48].

## Somatosensory evoked potentials (SSEPs)

SSEPs are valuable neurophysiological tools to assess subcortical and cortical integrity in comatose patients after CA [5,46,64]. Bilateral absence of the N20 cortical wave in SSEPs of the median nerves, recorded from 24 h after CA, is one of six prognostic markers already recommended for poor outcome prediction [7]. Indeed, a bilaterally absent N20 SSEP (defined as no responses or a response with an amplitude <0.20 μV according to recommendations [65]) is considered to be the most robust predictor of poor outcome. This criterion is highly specific (specificity 100%, FPR 0%) despite a moderate sensitivity (30–65 %, according to studies). Interestingly, the SSEPs N20 responses are not affected by usual doses of sedative drugs in the post-CA context [65]. However, N20 results could be affected by hypothermia (<35 °C), and low blood-pressure (mean arterial pressure – MAP <60 mmHg) [65].

Regarding the prognostic value of the bilateral N20 absence in the first 12−24 h post-CA [5,5,47,[66], [67], [68]], the specificity appears to approach 100%, comparable to that observed with recording performed after 24 h. Nevertheless, some rare cases have been reported of patients with bilateral absence of N20 at this early stage who subsequently showed a favourable outcome at 3 months [5]. In some cases, the cause of a false-positive result was an incorrect reading of the SSEP records [69]. Studies assessing interobserver agreement of SSEP reading have shown that the risk of misclassification is higher in the presence of artefacts or low amplitude of the N20 wave [70,71]. Thus, interrater agreement among SSEP readers increases significantly after neuromuscular blockade. Moreover, a recent study suggested that N20 SSEPs may provide high prognostic accuracy as early as 6 h after CA, possibly because they allow the assessment of a larger proportion of patients before their progression towards awakening or death [66]. Regarding late SSEP recording (i.e., after 4–6 days), data remain limited, but some studies suggested that the bilateral absence of N20 remains an excellent predictor of poor outcome [5].

Regarding the amplitude of the N20 wave, some studies have shown that a very low bilateral amplitude of the N20 (<0.40, 0.60 or 1 μV according to studies) is as specific as its bilateral absence in predicting a poor outcome, including when recorded at the very early phase (6−12 h post CA) [5,[66], [67], [68],[72], [73], [74], [75], [76]]. Conversely, the preservation of a bilateral N20 responses with high amplitudes (>3, 4 or 5 μV across studies) may be associated with favourable outcomes [4,68,[72], [73], [74], [75], [76]]. The prognostic value of the N20 amplitude may evolved over time after CA. First, the prognostic value of high N20 amplitudes for favorable outcomes could decreases between 12 and 72 h after CA, due to a progressive increase in N20 amplitudes among patients with poor outcomes [68]. Second, initially present but low-amplitude N20 responses may progressively disappear in patients with poor outcomes [67]. As N20 amplitude is strongly correlated with the severity of HIBI [77], these findings highlight the dynamic nature of HIBI and the potential evolution of N20 responses over time.

The prognostic value of a unilateral absence of N20 for predicting poor outcome remain also discussed. Two studies suggested that unilateral absence with a preserved but low voltage contralateral N20 response is highly specific of poor outcome, when recorded at 12, 24 or 72 h after CA [47,68]. Another study assessed the prognostic value of an unilateral N20 absence at the acute (i.e., 72 h after CA) and sub-acute phase (day 20 after CA): unilateral absence of N20 was highly specific of poor outcome (specificity 96.7%) despite very low sensitivity [78].

Finally, data concerning the N20 amplitude and unilateral absence of N20 are currently scarce, explaining why the ERC-ESICM 2025 recommendations do not recommend their use for assessing neurological prognosis after CA. Thus, further evidence is needed before these criteria can be incorporated into prognostic algorithms [7].

## Brain imaging

Brain imaging remains a major tool for neuroprognostication of unconscious patients after CA. Current 2025 ERC-ESICM guidelines recommend the use of either CT (at any time) or MRI (after 48 h but ideally before day 7) in the multimodal strategy [7]. Although significantly less sensitive than MRI, CT is frequently performed in the early phase after CA, to investigate the underlying cause of CA [7]. CT scan may be informative when early signs of poor outcome are observed, such as loss of differentiation between grey and white matter (using low values of G/W matter ratio, for example) or cerebral oedema [5,7]. However, because the optimal timing for detecting these abnormalities is beyond 48 h, CT performed at admission as part of the etiological assessment may be insufficiently sensitive to highlight these signs. Thus, the 2025 ERC-ESICM guidelines recommend repeating brain CT at 72−96 h from CA if the patient is still unconscious and the early brain CT does not show signs of HIBI. Otherwise, a normal CT scan is not systematically associated with a good outcome. In patients with an unremarkable CT, brain MRI should be considered, ideally beyond 48 h and within the first 7 days, when diffusion-weighted imaging (DWI) abnormalities are most reliably detected [5]. Indeed, MRI may help to predict poor and good outcomes, and remain more sensitive compared to CT. The 2025 ERC-ESICM recommendations suggested to consider “diffuse and extensive anoxo-ischemic injury” (i.e., diffuse and bilateral cortical and basal ganglia injuries) observed on the DWI sequence as highly specific of poor outcome [5,7]. Conversely, a normal DWI sequence in the first 7 days seems to be highly predictive of good outcome [4,7,9,79].

### Time effect on the prognostic value of MRI

However, MRI findings evolve over time and across brain regions, which may result in potential misinterpretation and misclassification (Fig. 3). Although the data are not very robust, it appears that the timing of MRI acquisition is crucial to ensure the detection of abnormalities predictive of poor outcome:

- Within the first 24 h, brain MRI may appear normal, including in patients with a poor outcome at 3 months [80].
- Between 48 h and day 7, MRI may begin to reveal cytotoxic oedema. This leads to restricted extracellular water diffusion, typically manifesting as DWI hyperintensity with corresponding apparent diffusion coefficient (ADC) reduction (ADC values < 0.6 × 10^−^³ mm²/s). The affected regions often include the cortical area and particularly the posterior cortical ribbon, the striatum, the splenium of the corpus callosum, and the hippocampi [81]. In this acute phase, FLAIR sequences may also demonstrate hyperintensity and thickening of cortical ribbon and basal ganglia. At this stage, the presence of extensive lesions (DWI hyperintensity) in all the cerebral cortex and basal ganglia is highly specific of poor outcome. Nevertheless, the assessments remaining potentially subjective and lacking a clearly established quantitative framework.
- After day 7, the subacute phase is characterized by vasogenic oedema, which appears hyperintense on both FLAIR and DWI, accompanied by a normalization of the ADC map (loss of diffusion restriction) [80]. Reduced DWI within the corona radiata may also appear, especially in patients with poor outcome, indicating the onset of white matter degeneration secondary to diffuse cortical injury. Finally, micro-haemorrhagic changes due to blood-brain barrier disruption typically emerge later in this phase on the susceptibility-weighted imaging (SWI). At this time, diffusion tensor imaging (DTI) could provide additional insight into white matter microstructure. Although of limited accessibility, some data suggested that reduced fractional anisotropy (FA) and increased diffusivity, markers of microstructural damage derived from DTI, are strongly correlated with functional outcome [82,83].
- After day 14, FLAIR imaging may be normal or demonstrate residual hyperintensities in some regions (cortical area, basal ganglia, and/or bilateral white matter), sometimes accompanied by cortical atrophy or heterogeneous signals due to necrosis. At this stage, DWI typically appears normal due to resolution of cytotoxic oedema, thus offering limited prognostic value [80,84]. SWI may show necrotic-haemorrhagic lesions, which are more frequent in this phase. Cortical or basal ganglia contrast enhancement on T1-weighted imaging after gadolinium administration could also be observed, related to blood–brain barrier disruption, and suggesting laminar necrosis. This criterion seems to be associated with poor outcome, although the data remain limited [80].

**Fig. 3:**
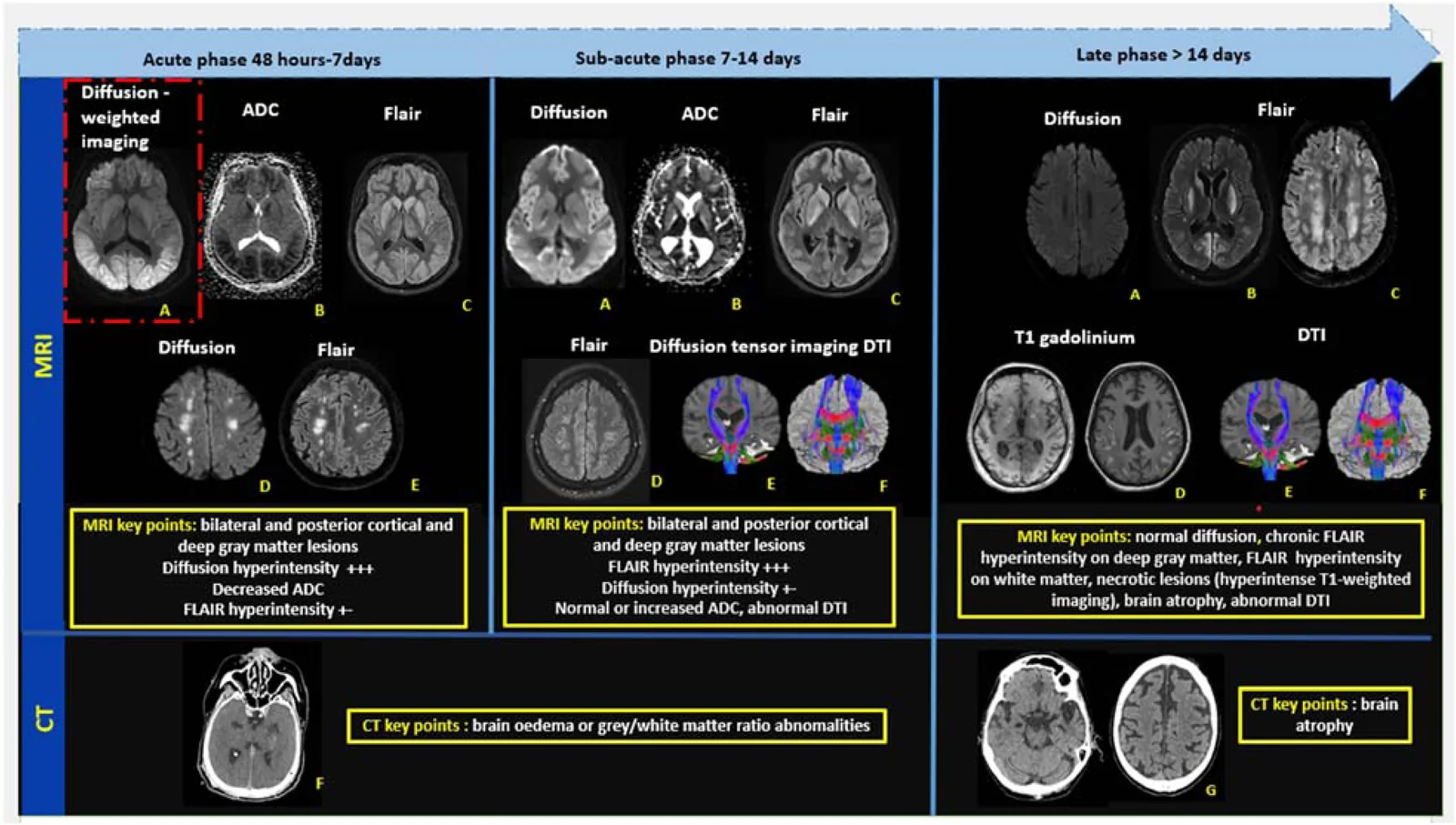
**Evolution of brain imaging (MRI or CT) according to time in comatose patients after cardiac arrest. The red box indicated the criteria considered as highly specific of poor outcome according to 2025 ERC-ESICM recommendations**. **Left part**: Extensive posterior cortical and deep gray matter lesions showing diffusion-weighted imaging (DWI) hyperintensity (A) with restricted diffusion on the apparent diffusion coefficient (ADC) map (B) and FLAIR hyperintensity (C). Multiple ischemic lesions in the centrum semi-oval (watershed territory), hyperintense on DWI (D) and FLAIR sequences (E), suggestive of hypoperfusion-related ischemia. CT scan showing brain oedema (F). **Central part**: moderate diffusion hyperintensity (A), diffuse increase in cortical and deep gray matter ADC values (B), diffuse cortical FLAIR hyperintensity (C, D); Diffusion tensor imaging (DTI)-based tractography in a patient with poor outcome (E) and a healthy control (F), showing a significant loss of white matter tracts. CT scan showing brain oedema (F). **Right part**: Normal or sub-normal DWI sequence (A), FLAIR hyperintensities in the cortico-subcortical regions and basal ganglia (B), FLAIR hyperintensity in the white matter (C), extensive necrotic lesions in the cortex and basal ganglia, appearing hyperintense on T1-weighted imaging, associated with significant cortical atrophy (D), DTI-based tractography in a patient with poor outcome (E) and a healthy control (F), showing a significant loss of white matter tracts. CT scan showing significant brain atrophy (G). *MRI: magnetic resonance imaging; CT: computed tomography* (For interpretation of the references to colour in this figure legend, the reader is referred to the web version of this article)*.*

## Biomarkers

Blood biomarkers of neuronal and glial injury are very useful for assessing neurological prognosis in comatose patients after CA (Fig. 4**)**. Currently, only serum neuron-specific enolase (NSE) is recommended by the 2025 ERC-ESICM guidelines to assess both favourable and unfavourable neurological outcomes. Indeed, a blood NSE level >60 μg/L at 48 and/or 72 h appears to be highly specific for predicting poor outcome [7]. An increasing NSE trend between 24, 48, and/or 72 h is also associated with an unfavourable outcome [7,[85], [86], [87]]. Conversely, low plasma NSE levels at 24, 48 and/or 72 h are highly predictive of a favourable outcome [7], as is a decrease in NSE levels over the first 72 h after CA [4,7,36,[85], [86], [87]]. Regarding the best threshold of NSE, current guidelines suggest considering NSE levels <17 μg/L within the first 72 h after CA as a highly specific, although poorly sensitive, predictor of favourable outcome [7]. Other studies have identified thresholds of NSE <30 or <45 μg/L within the first 72 h as providing the optimized balance between (higher) sensitivity and (slightly lower) specificity [36,45,85]. Thus, the currently recommended threshold may need to be reassessed and potentially raised, although further studies are warranted. In any case, low NSE levels combined with a decreasing trend over time should encourage intensivists to continue observation and reassess the patient's neurological status rather than making premature prognostic decisions. Regarding NSE trends, the NSE peak occurs around 72 h after CA in patients with poor outcome, and as early as 24 h in those with favourable outcome. Data regarding the potential benefit of measuring NSE beyond the very first days remain limited, and no clear threshold has been identified for values ​​obtained from 96 h onwards [4,5]. Therefore, late NSE measurements appear to be of limited utility. However, if measurements could not be performed within the first three days and a late value exceeds 60 μg/L, it may still be considered suggestive of poor outcome, given the known temporal kinetics of this biomarker [5,85]. Regarding NSE, several confounding factors must be considered: for example, haemolysis (for example, related to ECMO or other extracorporeal circuits) may increase serum NSE levels, or neuroendocrine tumours.

**Fig. 4:**
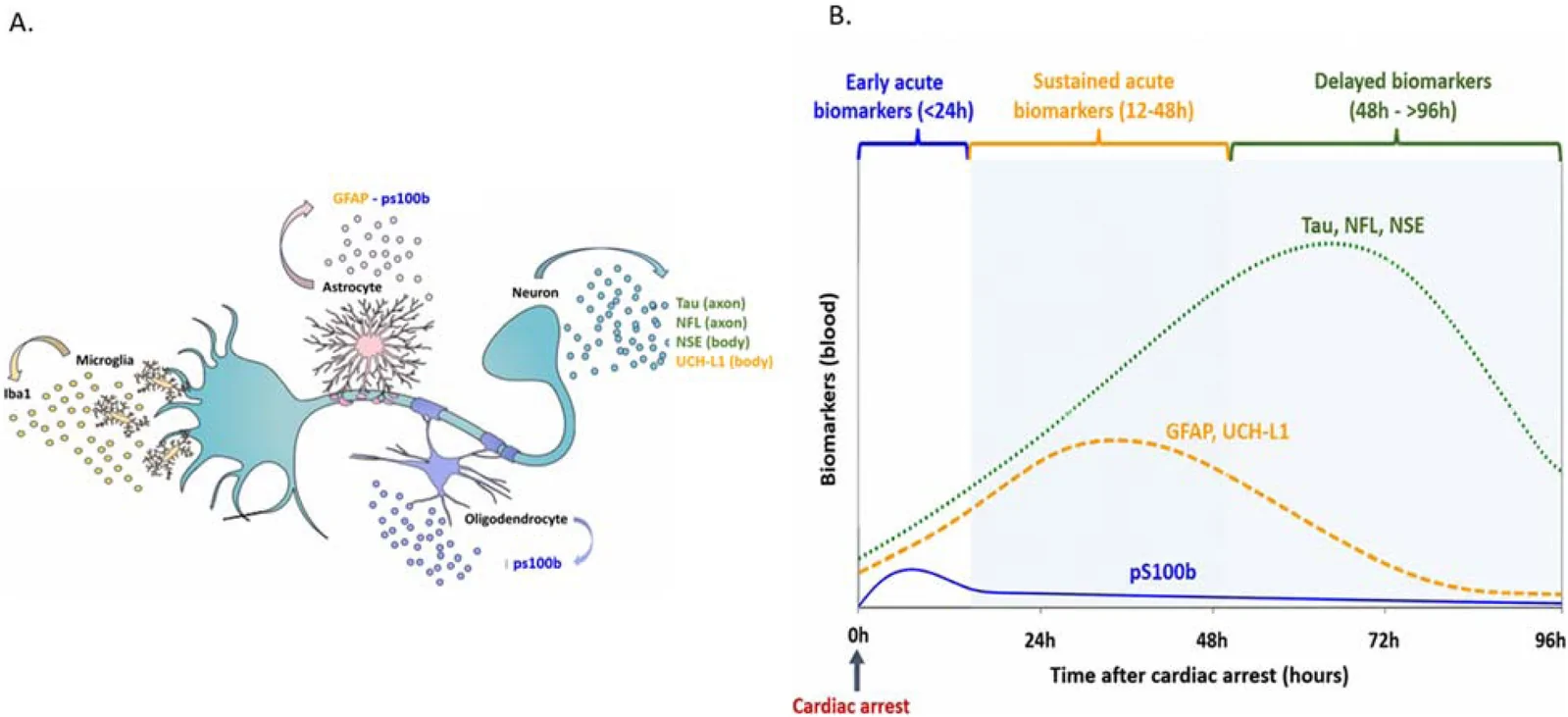
**Biomarkers of cellular damage (A, adapted from Benghanem et al, Resuscitation 2023), and kinetic profiles of each biomarkers (B, adapted from Lascarrou and Benghanem Resuscitation 2026) in comatose patients after cardiac arrest**. For Neuron Specific Enolase (NSE), an increasing trend between 24, 48, and/or 72 h is highly specific for poor neurological outcome at 3 months; conversely, a decreasing NSE trend over time is predictive of a favorable outcome. The kinetics of neurofilament light chain (NFL) over time have no prognostic value, as levels increase in both poor and good outcome groups. The kinetics of S100B, UCH-L1, GFAP, and Tau protein also have no prognostic value, as their levels decrease over time in both groups. *GFAP: Fibrillary Acidic Protein; NFL: neurofilament light chain; NSE: neuron-specific enolase; pS100b: S100 beta protein.*

Regarding other biomarkers of neuronal (TAU protein, Ubiquitin C-terminal Hydrolase L1- UCH-L1, and neurofilament light chain - NFL) or glial injury (S100B protein – S100Bp, Glial Fibrillary Astrocyte Protein - GFAP), the available data are more limited and less robust. They are not currently recommended for clinical practice, as cut-off values associated with good or poor outcomes are not well established [4,5]. Regarding their kinetics, TAU protein and NFL appear to reach a peak around 72 h after CA, similar to NSE, but their temporal evolution does not seem to have major prognostic value [87]. However, available data remain very limited, and further studies are needed to characterize the temporal kinetics of NFL levels (CINEFIL study, ClinicalTrials.gov identifier: NCT07760636). S100Bp is the earliest biomarker, peaking around 8 h after CA and within the first 24 h. Finally, GFAP and UCH-L1 appear to peak between 12 and 48 h after CA, although data remain scarce [87] (Fig. 4). Some recent data suggest that the prognostic performance of NFL (Area Under Curve - AUC 0.90–0.95) [85,[88], [89], [90], [91], [92], [93]] may be superior to that of NSE (AUC 0.80–0.90) at 24, 48, and 72 h [35,36,85,88,89,91,92]. However, the cut-off values of NFL for predicting good or poor outcomes are not yet well defined, and variability in assay techniques may explain the wide discrepancies in reported thresholds across studies) [85,[88], [89], [90], [91], [92], [93]]. Additional data are therefore needed to establish reliable cut-offs before considering NFL in clinical practices.

To conclude this review, we have summarized the prognostic performance of the different markers in terms of sensitivity, specificity and FPR, taking into account their evolution over time (Table 2).

**Table 2:** Prognostic performance of markers according to timing of assessment, in unconscious patients after cardiac arrest.

POOR OUTCOME				
Prognostic markers	Recommended timing of assessment	FPR (poor outcome) in the majority of studies	Sensitivity in the majority of studies	Main confounders / additional information
EEG suppression / burst-suppression (highly malignant)	≥24 h after CA	0%	Decreases over time, approximately 50%, 30% and 20% at 12, 24, and 36 h after CA; decreasing to 10% after day 7	Sedation; hypothermia; timing of recording; subjective interpretation
Bilateral absence of N20 SSEPs	≥24 h	0%	Relatively stable over time, approximately 30–65%	Potential technical artifacts (use neuromuscular blockade if needed), hypothermia (<35 °C), and low blood pressure (MAP < 60 mmHg).
Status myoclonus (generalized, continuous myoclonic movements persisting for more than 30 min)	≤72 h	0%	Relatively stable when observed during the first 72 h, approximately 5–40%	Sedation; neuromuscular blockade; challenging recognition and differential diagnosis (tremors, shivering, etc.)
Bilateral absence of pupillary and corneal reflexes	≥ 72 h	0%	Relatively stable over time, approximately 20%	Sedation/analgesia; neuromuscular blockade; difficulties in assessing the PLR in patients with pinpoint pupils (consider automated pupillometry)
MRI: diffuse, extensive hypoxic–ischemic injury on diffusion-weighted MRI	Ideally between days 2–7	0%	Relatively stable between days 2 and 7, approximately 35–90%; Low sensitivity before day 2; Progressive decrease in sensitivity after day 7	Timing; subjective interpretation, technical artifacts
CT: - Diffuse cerebral edema on CT - Marked reduction in the gray matter-to-white matter (GM/WM) ratio	- oedema: at any time (ideally between days 2–7) - GM/WM ratio: on brain CT ideally within 72 h	0%	Relatively stable between days 2 and 7, approximately 10−50%; Low sensitivity before day 2; Progressive decreasing of sensitivity after day 7	Timing; subjective interpretation, technical artifacts. For GM/WM ratio: region of interest (ROI) selection; threshold of GWR; timing of assessment
NSE > 60 μg/L and/or an increasing trend over time	48 and/or 72 h	0%	Relatively stable, approximately 40–80%; Progressive decrease in sensitivity after day 3	Hemolysis; assay differences; extracerebral sources

INDETERMINATE OUTCOME				
Prognostic markers	Recommended timing of assessment	FPR (poor outcome prediction) in the majority of studies	Sensitivity in the majority of studies	Main confounders / additional information
Intermediate EEG patterns (malignant)	>24 h	0–33.3%	Highly heterogeneous according to each EEG pattern	Sedation; hypothermia; timing of recording; subjective interpretation
Unilateral absence of N20 SSEPs	≥24 h	0−5%	Approximately 10%, limited data regarding temporal evolution	Potential technical artifacts (use neuromuscular blockade if needed), hypothermia (<35 °C), and low blood pressure (MAP < 60 mmHg), history of middle cerebral artery territory stroke.
Low-amplitude (< 0.40, 0.60 or 1 μV depending on the study) N20 SSEPs	≥24 h	0% but need confirmation	Relatively stable over time, approximately 40−65%	Potential technical artifacts (use neuromuscular blockade if needed), hypothermia (<35 °C), and low blood pressure (MAP < 60 mmHg).
Early isolated myoclonus	≤72 h	0–22%	Relatively stable over time, approximately 30–40%	Sedation; neuromuscular blockade; difficult recognition and differential diagnosis (tremors, shivering, etc.)
Absent corneal reflexes	≥ 72 h	0–7.7%	Relatively stable over time, around 25–50%	Sedation; neuromuscular blockade
Absent pupillary light reflexes	≥ 72 h	0–7.7%	Relatively stable over time, around 20−35%	Sedation/analgesia; neuromuscular blockade; difficulties in assessing the PLR in patients with pinpoint pupils (consider automated pupillometry)

FAVORABLE OUTCOME				
Prognostic markers	Recommended timing of assessment	Specificity (good outcome prediction) in the majority of studies	Sensitivity in the majority of studies	Main confounders / additional information
GCS motor score 4–5	≤96h	84–98%	Approximately 10–75%; Not recommended after 96h	Sedation; neuromuscular blockade
Continuous / nearly continuous normal-voltage EEG without discharges (if reactive: benign EEG)	≤72 h	56.5–100%	Relatively stable in the first 72 h, approximately 30–80%	Sedation; hypothermia; timing of recording; potentially subjective interpretation
Normal diffusion-weighted MRI	Ideally between Day 2 and Day 7	60–95%	Approximately 50–100%; Decreased after day 7	Timing; subjective interpretation, technical artifacts
High N20 amplitude (>3–4 μV)	≥ 24 h	85–95%	Approximately 10–60%; May decreased over time	Potential technical artifacts (use neuromuscular blockade if needed), hypothermia (<35 °C), and low blood pressure (MAP < 60 mmHg).
Low NSE (<17 μg/L) +- descending trends over time	Within 72 h	80–96%	Approximately 26−57%; Not recommended after 72 h	Hemolysis; assay differences; extracerebral sources
Low NSE (<30 or <40–45 μg/L) and/or a decreasing trend over time	Within 72 h	86%	Around 68−100%; Not recommended after 72 h	Hemolysis; assay differences; extracerebral sources

Data presented in this table are mainly derived from two systematic reviews by Sandroni et al. on predictors of poor neurological outcome (Intensive Care Medicine, 2020) and favorable neurological outcome (Intensive Care Medicine, 2022), as well as the 2025 ERC–ESICM guidelines on post-resuscitation care (Nolan et al., Intensive Care Medicine/Resuscitation, 2025). *CA, cardiac arrest; CT, computed tomography; EEG, electroencephalography; FPR, false-positive rate; GCS, Glasgow Coma Scale; MRI, magnetic resonance imaging; NSE, neuron-specific enolase; ROI, region of interest; SSEP, somatosensory evoked potential.*

## Conclusion

Available data confirms that clinicians now have clinical and paraclinical tools with well-established prognostic value for predicting neurological outcomes in comatose patients following CA. Combined within a multimodal algorithm, their performance has been confirmed in various clinical studies. These data also confirm the critical importance of strictly adhering to the recommended timing for collecting these parameters, as their interpretation and prognostic value can vary considerably over time. Future clinical research efforts should continue to strengthen this strategy, particularly by examining the added value of new indicators (biomarkers, late auditory evoked potentials, brain imaging techniques such as functional MRI, etc.) and testing them in clinical trials that minimize the risk of bias.

## Authors' contributions

Concept and design: SB, AC, CL. Drafting of the manuscript: all authors; Critical revision of the manuscript for important intellectual content: all authors. Pr Alain Cariou is a member of the editorial board of Annals of intensive Care.

## Consent for publication

Not applicable.

## Ethics approval and consent to participate

Not applicable.

## Declaration of Generative AI and AI-assisted technologies in the writing process

The authors used ChatGPT (OpenAI) solely for English-language editing and grammatical checking. All content was reviewed and verified by the authors, who take full responsibility for the final manuscript.

## Funding

Not applicable

## Availability of data and material

Not applicable

## Declaration of competing interest

The authors declare that they have no competing interests. Pr Alain Cariou is a member of the editorial board of Annals of intensive Care.
