# Supplementary material for: Neuroprognostication in unconscious patients after cardiac arrest: considering the time course of prognostic markers
Source: Ann Intensive Care. 2026 Oct 29;16:100195. doi: 10.1016/j.aicoj.2026.100195 (PMC13761240; doi:10.1016/j.aicoj.2026.100195)
Supplement: Supplementary file 1 [file mmc1.docx]

Table 1. Diagnostic criteria of clinical status of consciousness

| Clinical status | Delay | Brainstem reflexes | Auditory function | Visual function | Motor function | Oro-motor function | Communication | Arousal |
| --- | --- | --- | --- | --- | --- | --- | --- | --- |
| Coma | Early phase (<7 days) | Inconsistent | None | None | None | None | None | Unarousable |
| Vegetative state (VS)/ unresponsive wakefulness syndrome (UWS) | Sub-acut phase (>7 days) | Present | Auditory startle / Localization to sound | Visual startle (blink to threat) | Abnormal posturing / Flexion withdrawal | Vocalization / oral movement | None | Eye opening with and without stimulation |
| Minimal conscious state – (MCS) | Sub-acute phase (>7 days) | Present | Consistent movement to command / Reproducible movement to command | Visual pursuit / Object localization (reaching) / Object recognition | Localization to noxious stimulation / Object manipulation / Automatic motor response | Intelligible verbalization | Non-functional communication (intentional) | Attention |
| Exit - minimal conscious state (E-MCS) | Could be observed as an evolutive of MCS | Present | Consistent movement to command / Reproducible movement to command | Visual pursuit / Object localization (reaching) / Object recognition | Functional object use | Intelligible verbalization | Functional communication | Attention |
| Cognitive motor dissociation – CMD  (usually assessed with functional MRI or EEG) | At any time | Present | Auditory startle / Localization to sound | Visual startle / Visual pursuit / Object localization | Localization to noxious stimulation / Object manipulation / Automatic motor response | Vocalization / oral movement | None | Unarousable  Or Eye opening with and without stimulationOr attention |

***Figure 1. Illustration of EEG patterns in comatose patients after cardiac arrest.*** *The patterns are described according to the background rhythm, superimposed abnormalities, and reactivity. The correlation between EEG patterns and the severity of hypoxic ischemic brain injury (HIBI) is indicated by the arrow on the right. Definitions of EEG patterns are derived from the terminology of the American Clinical Neurophysiology Society (ACNS, Hirsch et al* [26]*). According to the classification of Westhall et al*[27]*, “highly malignant” patterns include suppression patterns (with or without superimposed periodic discharges) and burst-suppression patterns; “malignant” patterns include discontinuous background, low-voltage background, unreactive EEG, electrographic seizures, and abundant rhythmic or periodic patterns (RPPs); finally, “benign” patterns are defined by the absence of malignant or highly malignant features, corresponding to a continuous or nearly continuous background (discontinuity <10%), normal voltage (amplitude >20 µV), and preserved reactivity.*
